# Enhanced rates of enzymatic saccharification and catalytic synthesis of biofuel substrates in gelatinized cellulose generated by trifluoroacetic acid

**DOI:** 10.1186/s13068-017-0999-2

**Published:** 2017-12-27

**Authors:** Tânia M. Shiga, Weihua Xiao, Haibing Yang, Ximing Zhang, Anna T. Olek, Bryon S. Donohoe, Jiliang Liu, Lee Makowski, Tao Hou, Maureen C. McCann, Nicholas C. Carpita, Nathan S. Mosier

**Affiliations:** 1 Department of Botany & Plant Pathology, Purdue University, West Lafayette, IN 47907 USA; 2 College of Engineering, China Agricultural University, Beijing, 100083 People’s Republic of China; 3 Laboratory of Renewable Resources Engineering, Purdue University, West Lafayette, IN 47907 USA; 4 Department of Agricultural and Biological Engineering, Purdue University, West Lafayette, IN 47907 USA; 5 Department of Biological Sciences, Purdue University, West Lafayette, IN 47907 USA; 6 Biosciences Center, National Renewable Energy Laboratory, Golden, CO 80401 USA; 7 Department of Bioengineering, Northeastern University, Boston, MA 02115 USA; 8 Department of Chemistry and Chemical Biology, Northeastern University, Boston, MA 02115 USA; 9 Bindley Bioscience Center, Purdue University, West Lafayette, IN 47907 USA; 10 Present Address: Department of Food Science and Experimental Nutrition, University of São Paulo, Av. Prof. Lineu Prestes, 580, Bloco 14, São Paul, SP 05508-000 Brazil; 11 Present Address: Center for Functional Nanomaterials, Brookhaven National Laboratory, Shirley, New York, USA

## Abstract

**Background:**

The crystallinity of cellulose is a principal factor limiting the efficient hydrolysis of biomass to fermentable sugars or direct catalytic conversion to biofuel components. We evaluated the impact of TFA-induced gelatinization of crystalline cellulose on enhancement of enzymatic digestion and catalytic conversion to biofuel substrates.

**Results:**

Low-temperature swelling of cotton linter cellulose in TFA at subzero temperatures followed by gentle heating to 55 °C dissolves the microfibril structure and forms composites of crystalline and amorphous gels upon addition of ethanol. The extent of gelatinization of crystalline cellulose was determined by reduction of birefringence in darkfield microscopy, loss of X-ray diffractability, and loss of resistance to acid hydrolysis. Upon freeze-drying, an additional degree of crystallinity returned as mostly cellulose II. Both enzymatic digestion with a commercial cellulase cocktail and maleic acid/AlCl_3_-catalyzed conversion to 5-hydroxymethylfurfural and levulinic acid were markedly enhanced with the low-temperature swollen cellulose. Only small improvements in rates and extent of hydrolysis and catalytic conversion were achieved upon heating to fully dissolve cellulose.

**Conclusions:**

Low-temperature swelling of cellulose in TFA substantially reduces recalcitrance of crystalline cellulose to both enzymatic digestion and catalytic conversion. In a closed system to prevent loss of fluorohydrocarbons, the relative ease of recovery and regeneration of TFA by distillation makes it a potentially useful agent in large-scale deconstruction of biomass, not only for enzymatic depolymerization but also for enhancing rates of catalytic conversion to biofuel components and useful bio-products.

## Background

Lignin and the crystallinity of cellulose are considered major recalcitrance factors impeding the biological or chemical conversion of cellulose in biomass to biofuels or bio-based products [1, 2]. Cellulose microfibrils are long *para*-crystalline arrays of several dozen (1→4)-β-d-glucan chains with a degree of polymerization (DP) of up to 20,000 for secondary walls of lignocellulosic biomass [3–5]. Individual microfibrils synthesized at the plasma membrane surface are about 3 nm in diameter, but bundle into much larger macrofibrils of up to 30 nm or greater with crystalline continuity [6–8].

Mechanical disruption of the cellulosic source by ball milling can fragment macrofibrils and reduce crystallinity to a certain extent, thereby increasing sites of enzyme or catalyst accessibility [9–11]. Treatments with dilute acids improve enzymatic yields of fermentable sugars, but hydrolysis and loss of non-cellulosic sugars, decomposition of sugars at high temperatures, problems with acid recovery, and other environmental considerations prompted a search for alternatives [12–15]. Steam expansion at neutral temperatures reduced decomposition, and the separation of lignin and cellulose improved subsequent enzymatic digestion to fermentable sugars [16, 17]. Ammonia freeze-explosion (or fiber-expansion) (AFEX) pretreatment with high-pressure liquid anhydrous ammonia has been optimized for various biomass types to provide a clean stream of cellulose for fermentation and recovery of the ammonia [18–20]. AFEX swells biomass, enabling nearly complete enzymatic conversion of cellulose fermentable sugars [16]. The ammonia treatment extracts xylans and redistributes lignin to the surface of the cell walls, enhancing access of enzymes to cellulose through creation of large, porous networks [21]. Biomass treated with steam at high pressure and temperature alone is sufficient to enhance the yields of sugars from enzymatic digestion [22, 23].

Swelling of cellulose that occurs during either steam expansion or AFEX enhances final yield of sugar but does not enhance the time to completion, indicating that lignin interference with hydrolysis might be attenuated, but the crystallinity of cellulose remains a significant recalcitrance factor. Several classic methods have been employed to solubilize cellulose for improved saccharification yield, such as combinations of NaOH/urea or 85% phosphoric acid [24], but they are not without problems with cellulose decomposition or depolymerization and recovery of reagents. Molten ionic liquids [25], such as 1-butyl-3-methylimidazolium (BMIM) chloride or 4-methylmorpholine 4-oxide (NMMO), solubilize cellulose at relatively low temperatures without inducing extensive modification [26–28]; the interaction of imidazolium-containing ILs better dissolves lignin in addition to generating amorphous cellulose from lignocellulosic biomass [29–31]. Because addition of water or ethanol results in precipitation of the cellulose, the ILs can be recovered [32, 33], and the decrystallized cellulose exhibits strongly enhanced enzymatic digestibility [28, 33]. Although ILs are favored as ‘green’ chemicals due to their low volatility and potentially low environmental impact, concerns remain about expense of the reagents and their recovery and regeneration costs at commercial scale [32, 34]. In addition to substantial improvements in biodigestibility of cellulose, bacterial strains engineered to digest polysaccharide substrates can convert these digestion products into fuel molecules [35].

An alternative to ILs to dissolve cellulose is trifluoroacetic acid [36, 37]. Its activity is unusual in that freezing temperatures drive solubilization by penetration of TFA diesters into the cellulose microfibrils, with some TFA monoesters forming with glucosyl residues of the cellulose chains [37]. Gentle heating cleaves the esters and produces gelatinized forms of cellulose. As with ionic liquids, decrystallized cellulose is recovered as a gel upon addition of an alcohol such as isopropanol or ethanol [38]. Our study here aimed to characterize the transition from crystalline to gelatinized cellulose, and to assess the minimum treatment needed to improve enzyme digestibility and chemical catalysis to biofuel molecules. We report here that partial dissolution of cellulose by subzero temperatures is sufficient to substantially enhance both enzymatic digestion and catalysis to fuel substrates.

## Results

### TFA solubilization and generation of gelatinized cellulose

**Fig. 1:**
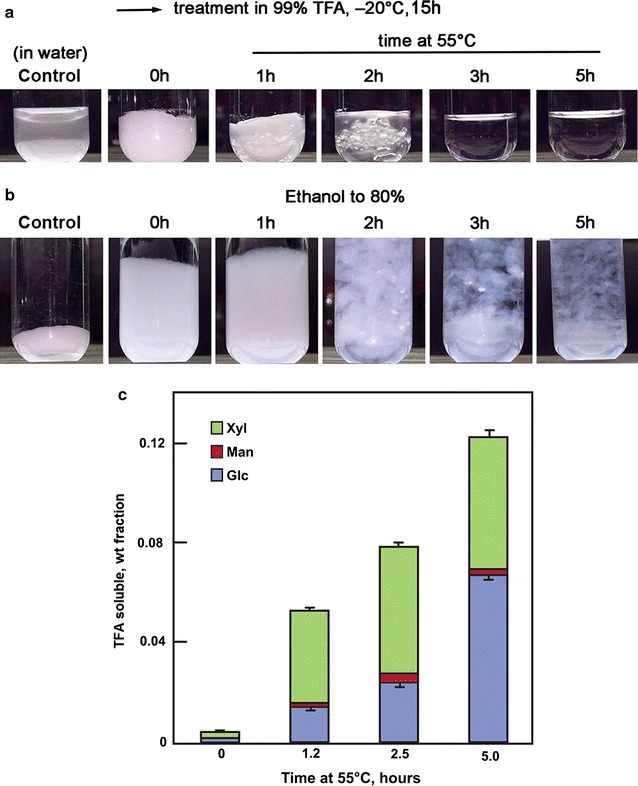
Physical behavior of cotton linter cellulose in TFA. **a** Dry cotton linter cellulose powder was suspended in TFA at − 20 °C and incubated for 15 h at that temperature. Samples were rapidly suspended by vortex mixing in five volumes of ethanol at ambient temperature (0 h) or heated to 55 °C for up to 5 h before addition of ethanol. **b** Appearance of cellulose in each of the treatments after several washes in 80% ethanol in water (v/v), and in water. **c** Weight fraction of TFA-soluble carbohydrate. Mole% of major monosaccharides xylose, glucose, and mannose were determined as alditol acetates by GC–MS [38, 55]

Cotton linter cellulose (50 mg mL^−1^) maintained at − 20 °C for 15 h formed a thick, semi-solid slurry that melted into a clear solution between 2 and 5 h of subsequent incubation at 55 °C (Fig. 1a). Upon addition of five volumes of ethanol, the appearance of the mixture differed on the extent of heating, from a swollen opaque gel without heating to translucent gels after about 2.5 h at 55 °C (Fig. 1b). Similar behaviors were observed when reactions were scaled to 1 g of the cellulose in 30 mL of ice-cold TFA. These gelatinized forms persisted after several washes with 80% (v/v) ethanol and with water. Subsequent analyses were performed with either these ‘never-dried’ gels or paired samples that had been flash-frozen and lyophilized.

Monosaccharide analyses of cell wall material solubilized by the TFA treatment showed that very little material was lost from swollen cellulose, but heating resulted in gradual hydrolysis up about 12% weight fraction of the linter wall material by 5 h (Fig. 1c). Analysis of the soluble material after swelling showed that it was 56 mol% xylose and 44 mol% glucose, with a small amount of mannose. Upon heating at 55 °C, xylose was about 70 mol% of the soluble product after 1.2 h and decreased to 38 mol% after 5 h. Conversely, glucose increased from 26 to 48 mol% of the soluble fraction (Fig. 1c). The total mass of soluble xylose remains nearly constant over the heating period, likely because it is completely hydrolyzed within the first 1.2 h. The increase in total soluble product over extended heating is from the much slower hydrolysis of cellulose.

### Analysis of crystalline and gelatinized cellulose by microscopy

**Fig. 2:**
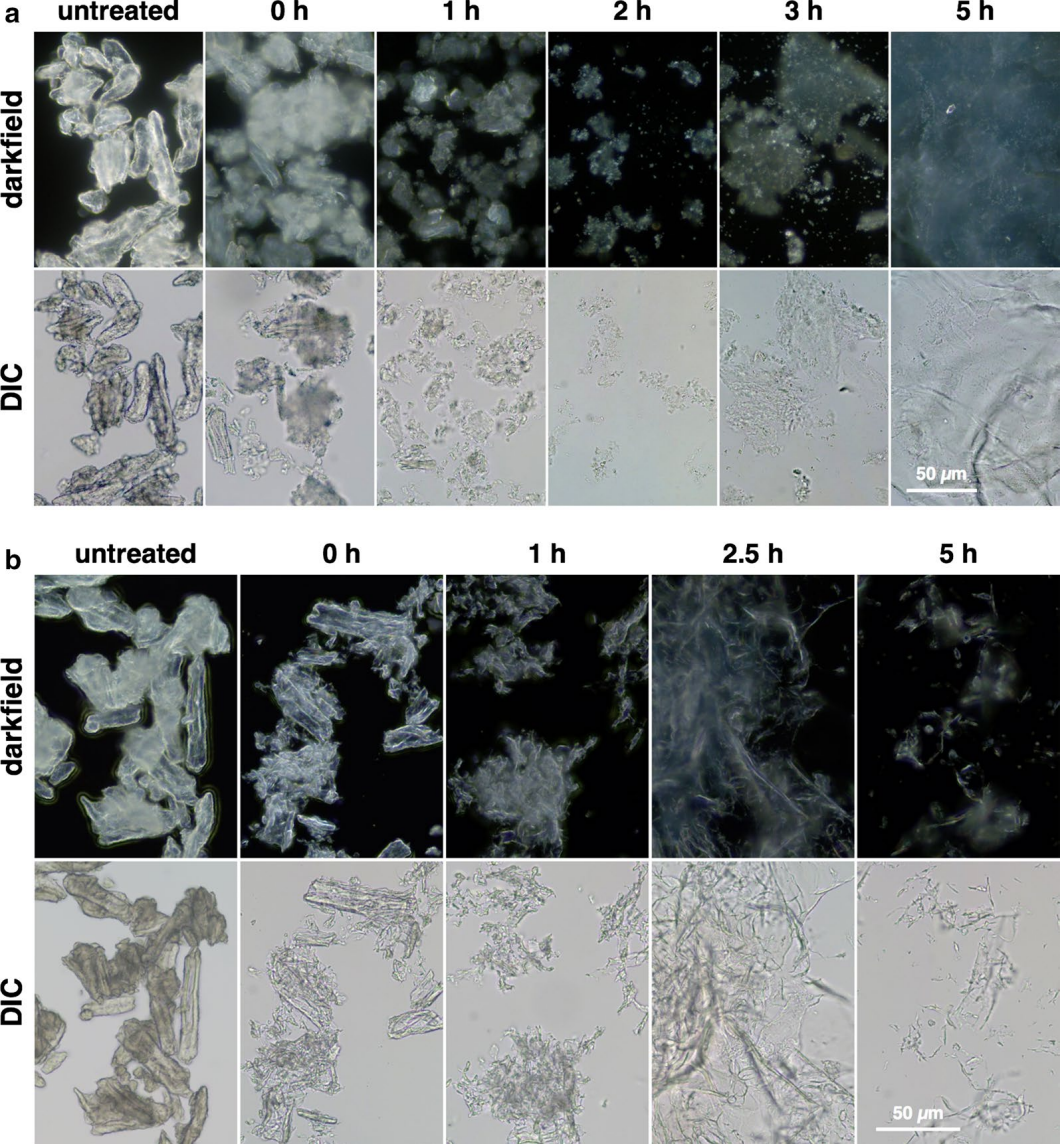
Loss of birefringence upon treatment with TFA as determined by darkfield microscopy (upper panels) compared to gel forms observed by differential interference contrast (DIC) microscopy (lower panels). **a** Never-dried samples in water. **b** Freeze-dried. Times are hours of heating at 55 °C following low-temperature swelling at − 20 °C for 15 h

**Fig. 3:**
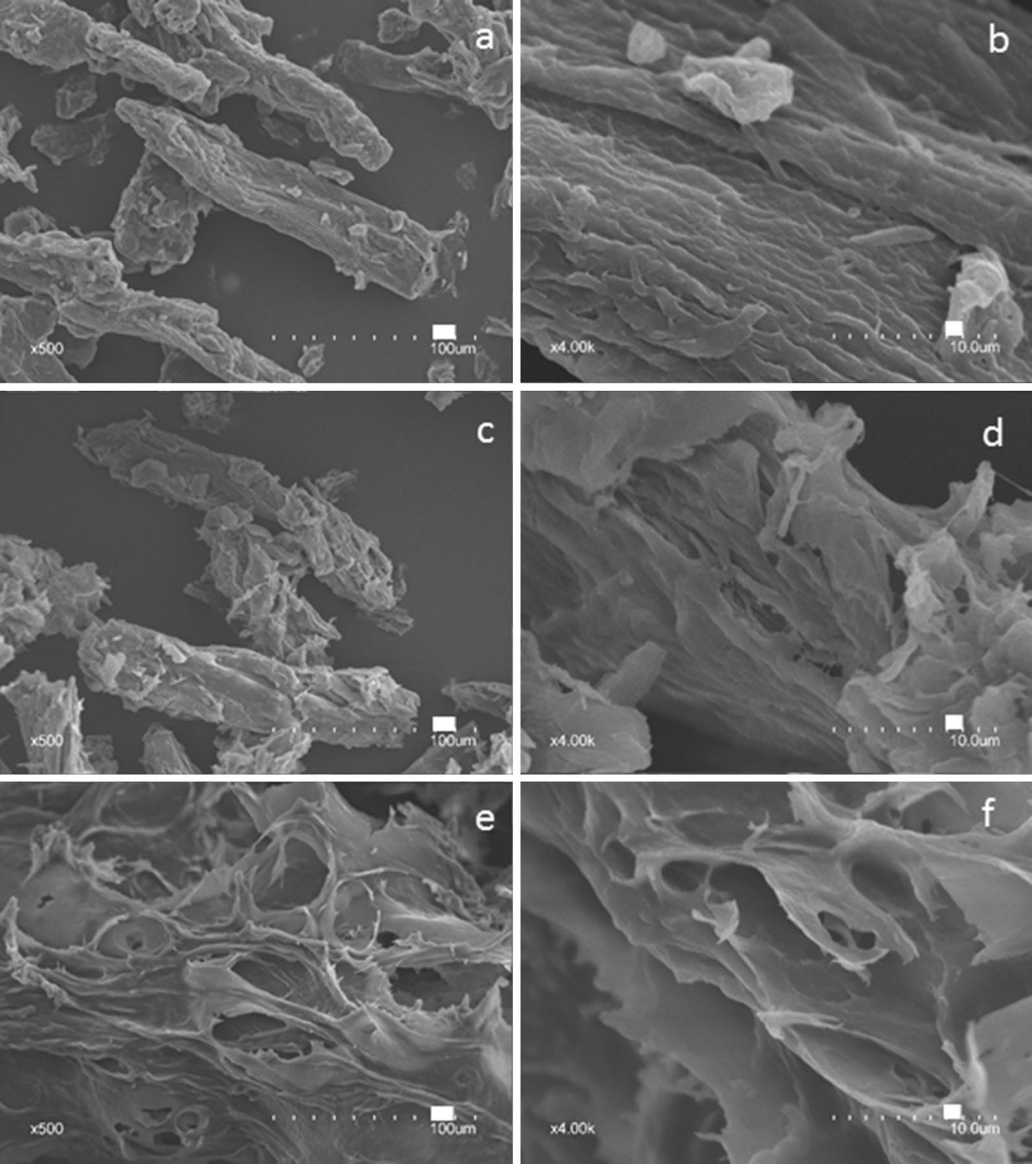
Changes in surface structure of cotton linter cellulose after low-temperature swelling and subsequent gelatinization at 55 °C. After each treatment, swollen and gelatinized materials were washed extensively in water and freeze-dried. **a** Untreated cotton fiber linter particles. Bar = 100 μm. **b** Higher magnification shows undulations in the surface of the fiber fragments. Bar = 10 μm. **c** Low-temperature swollen particles (− 15 °C for 24 h) show erosion and wrinkling of the surface. Bar = 100 μm. **d** Higher magnification shows potentially defibrillated macrofibrils. Bar = 10 μm. **e** Gelatinization of swollen fiber particles at 55 °C for 5 h results in loss of cellular integrity. Bar = 100 μm. **f** Higher magnification reveals loss of macrofibril structure. Bar = 10 μm

Darkfield microscopy showed the gradual loss of the characteristic intense birefringence of crystalline cellulose to a more diffuse type, indicating conversion to an amorphous form (Fig. 2a). Upon freeze-drying, a proportion of the gelatinized cellulose reannealed into crystalline forms, as seen by reappearance of sharper birefringence (Fig. 2b). Scanning electron microscopy (SEM) showed the cotton linter cellulose particles to be rod-shaped with diameters of about 10 μm (Fig. 3a), with undulations on the cell surfaces of 0.5–1 μm in diameter (Fig. 3a). Particles of similar dimensions were observed after low-temperature swelling with TFA for 2 h at 0 °C or for 24 h at − 15 °C, with partial erosion and wrinkling of the surface, and some fragmentation (Fig. 3c), but substantial dissociation of macrofibril structure was observed compared to untreated materials (Fig. 3d). Heating of the swollen particles to gelatinize cellulose resulted in complete loss of cellular integrity and macrofibril structure, and showed a transition to a fused-and-porous morphology with increased times of heating (Fig. 3e, f).

### Loss of thermostability and resistance to acid hydrolysis of gelatinized cellulose

**Fig. 4:**
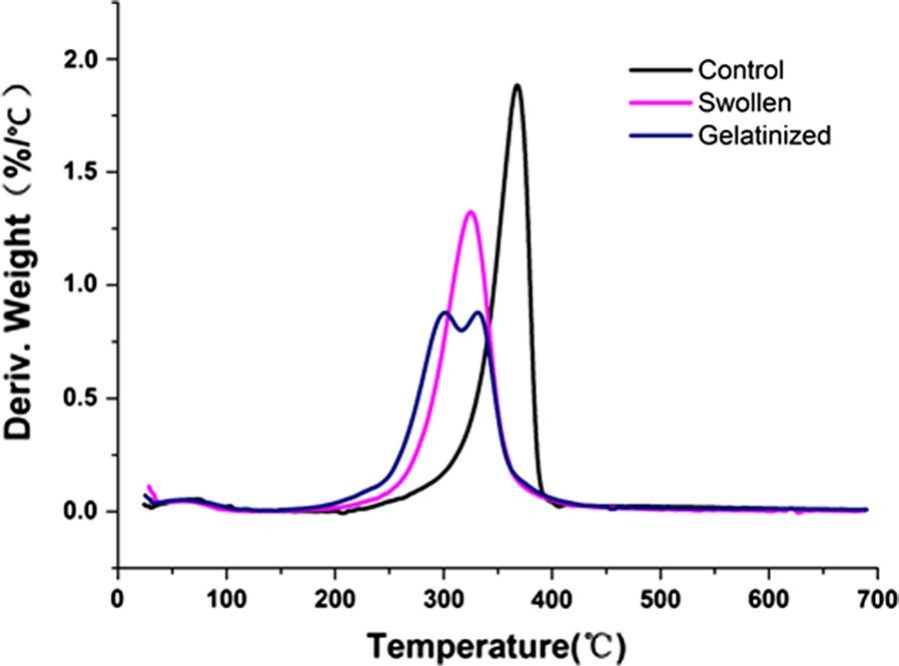
Differential thermogravimetry (DTG) biphasic behaviors of control, swollen, and gelatinized cotton linter cellulose. Cotton linter cellulose was low-temperature swollen at − 20 °C for 24 h. Swollen cellulose was gelatinized by heating at 55 °C for 5 h

Thermogravimetry (TG) measures changes in weight during heating, and derivative thermogravimetry (DTG) measures variation in the rate of weight change during heating. Cotton linter cellulose gave rates of weight change consistent with cellulose decomposition, with a homogeneous peak of principal decomposition step between 300 and 360 °C [39, 40]. The initial weight loss initiated at 50 °C was attributed to the evaporation of free water in the samples. Loss of thermostability in TFA treatments was biphasic. Compared to untreated cellulose, a portion of low-temperature swollen cellulose showed slightly lower onset of weight loss temperatures, indicating the development of a different state in addition to that characteristic of the crystalline form (Fig. 4). However, two broad and overlapping decomposition curves were observed with gelatinized cellulose; the first began at about 250 °C and reached a peak at 290 °C, and a second peak began about 310 °C followed by the main decomposition peak at 340 °C. The intensity of lower temperature decomposition profile increased with TFA gelatinization (Fig. 4).

**Fig. 5:**
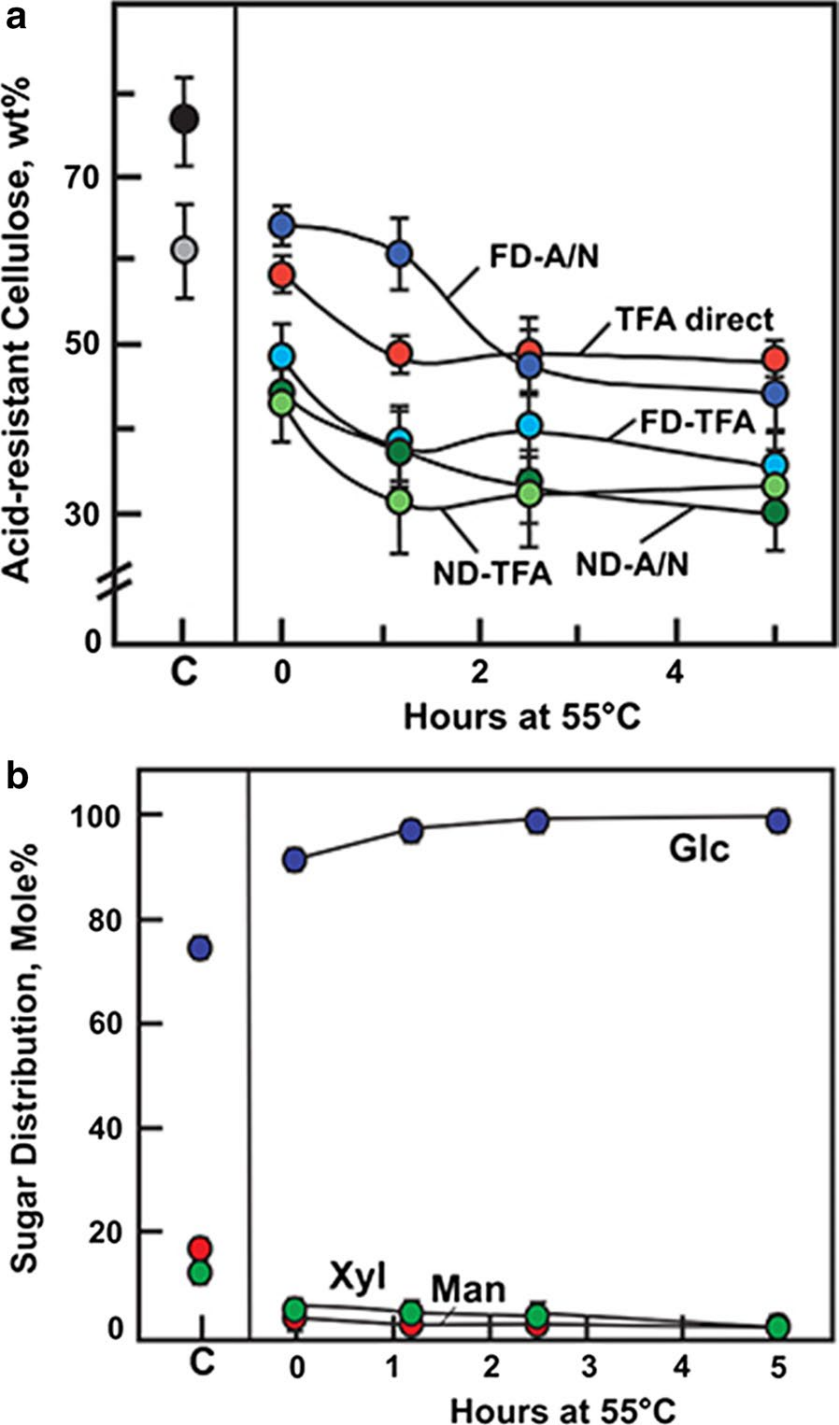
TFA hydrolysis of swollen and gelatinized cellulose generated by TFA. Cellulose samples were swollen in TFA at − 20 °C for 15 h and heated to 55 °C for up to 5 h before addition of four volumes of ethanol and washing in water. **a** 5 mg samples of freeze-dried (FD) and 0.5-mL suspensions of never-dried (ND) swollen and gelatinized cellulose were brought to 2-M TFA and heated to 120 °C for 90 min. Equivalent samples were hydrolyzed in acetic–nitric (A/N) reagent [38]. Control samples in TFA were diluted to 2 M with water and hydrolyzed at 120 °C (TFA direct). Additional controls were hydrolyzed with either 2 M TFA or A/N reagent. After hydrolysis, samples were centrifuged to pellet residual cellulose, and the TFA solutions dried under N_2_. Glucose equivalents were determined by a phenol–sulfuric assay [47, 57] in both TFA-soluble and insoluble fractions. Values represent mean and variance or S.D. of two to four samples. **b** Solubilized sugars from 2-M TFA hydrolysis at 120 °C were dried under a stream of nitrogen gas, and converted to alditol acetates for characterization by GC–MS [38, 55]. Values represent the means of two to four samples, with variance or S.D. within the width of the symbols

About 22% of the cotton linter cellulose was hydrolyzed by 2 M TFA at 120 °C for 90 min (Fig. 5a), a treatment commonly used in general hydrolysis of non-cellulosic polysaccharides to monosaccharides [41]. In contrast, over 50% of the low-temperature swollen cellulose and 60–70% of the gelatinized cellulose was hydrolyzed by 2 M TFA (Fig. 5a). Freeze-dried gelatinized cellulose was generally more resistant to hydrolysis than were the never-dried samples. About 60% of the cotton linter cellulose was resistant to an alternative acetic-nitric digestion method [42], and low-temperature swollen cellulose after freeze-drying was equally resistant (Fig. 5a). Freeze-dried gelatinized cellulose was hydrolyzed by the acetic-nitric reagent to slightly higher extents than did TFA (Fig. 5a). To determine the extent of resistance to acid hydrolysis that was imparted by precipitation in ethanol, cellulose was solubilized in TFA and diluted immediately to 2 M for hydrolysis at 120 °C for 90 min. Such treatment resulted in rapid precipitation and even higher resistance to hydrolysis (Fig. 5a). Of the insoluble material remaining after TFA treatment, the vast majority of the monosaccharide solubilized by acid hydrolysis was glucose, increasing from 91 mol% in the material from low-temperature swelling to over 98 mol% after treatment for 5 h at 55 °C (Fig. 5b).

### Relative loss in degree of polymerization during gelatinization

**Fig. 6:**
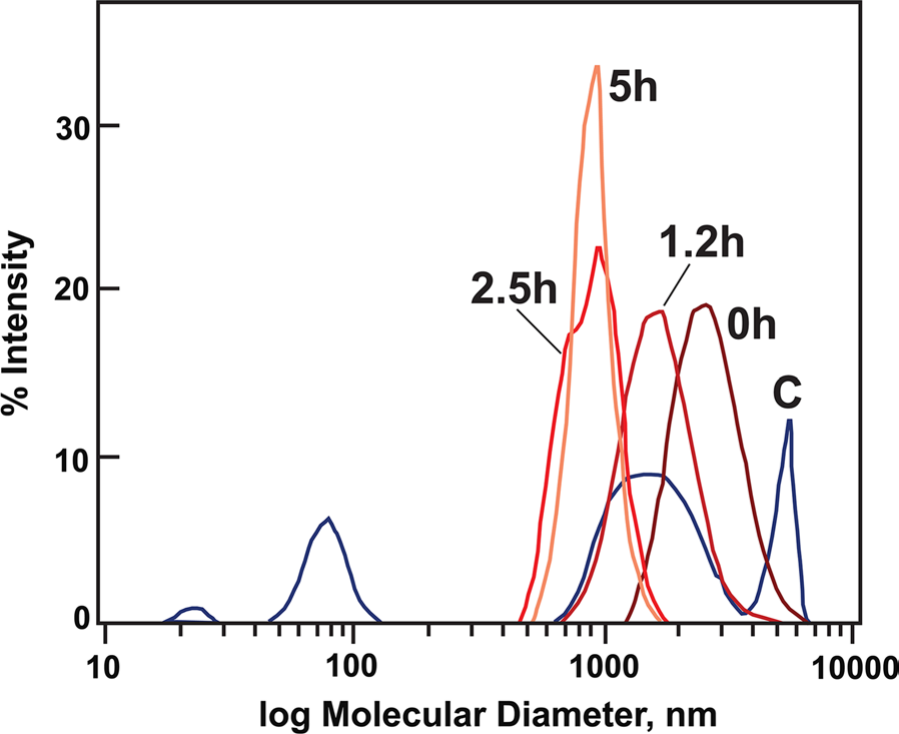
Relative molecular diameters determined by dynamic light scattering of cellulose after treatment with TFA. Freeze-dried TFA-treated and control celluloses were dissolved at 10 mg mL^−1^ in 4-methylmorpholino-4-oxide hydrate (NMMO) at 90 °C for 4 h with stirring. Analyses were made in a sample chamber held at 85 °C. *C* control cotton linter cellulose; Times in hours are incubations at 55 °C after swelling at − 20 °C for 15 h

Estimations of degree of polymerization (DP) by reducing end analysis compared to total mass or methylation analysis to determine the ratio of *t*-Glucose to 4-Glucose residues were compromised by the high proportion of non-cellulosic glucans in the cotton linter cellulose. Dynamic light scattering of control and TFA-solubilized gave relative molecular diameters after dissolution in NMMO. Cotton linter cellulose gave several polydisperse distributions, with one of the highest molecular diameter peaks centered at 5000–6000 nm, a second broad distribution of lower molecular diameters, and a third distribution of small diameter material. The lower molecular diameter materials were absent from all TFA-treated samples. Molecular diameters of swollen cellulose decreased to 3000 nm, and heating at 55 °C lowered diameters to less than 1000 nm (Fig. 6).

### Analysis of crystalline and amorphous cellulose content by X-ray diffraction and FTIR spectroscopy

**Fig. 7:**
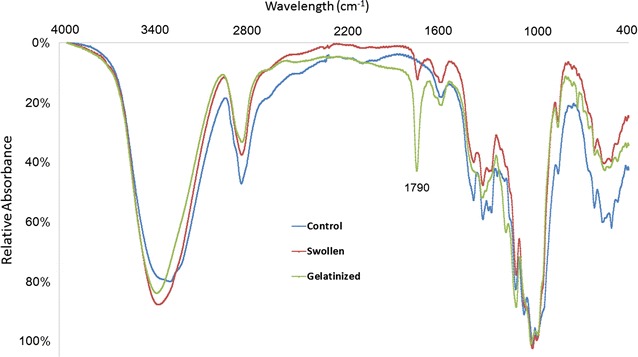
FTIR adsorption spectrum of TFA-treated and untreated cellulose

**Fig. 8:**
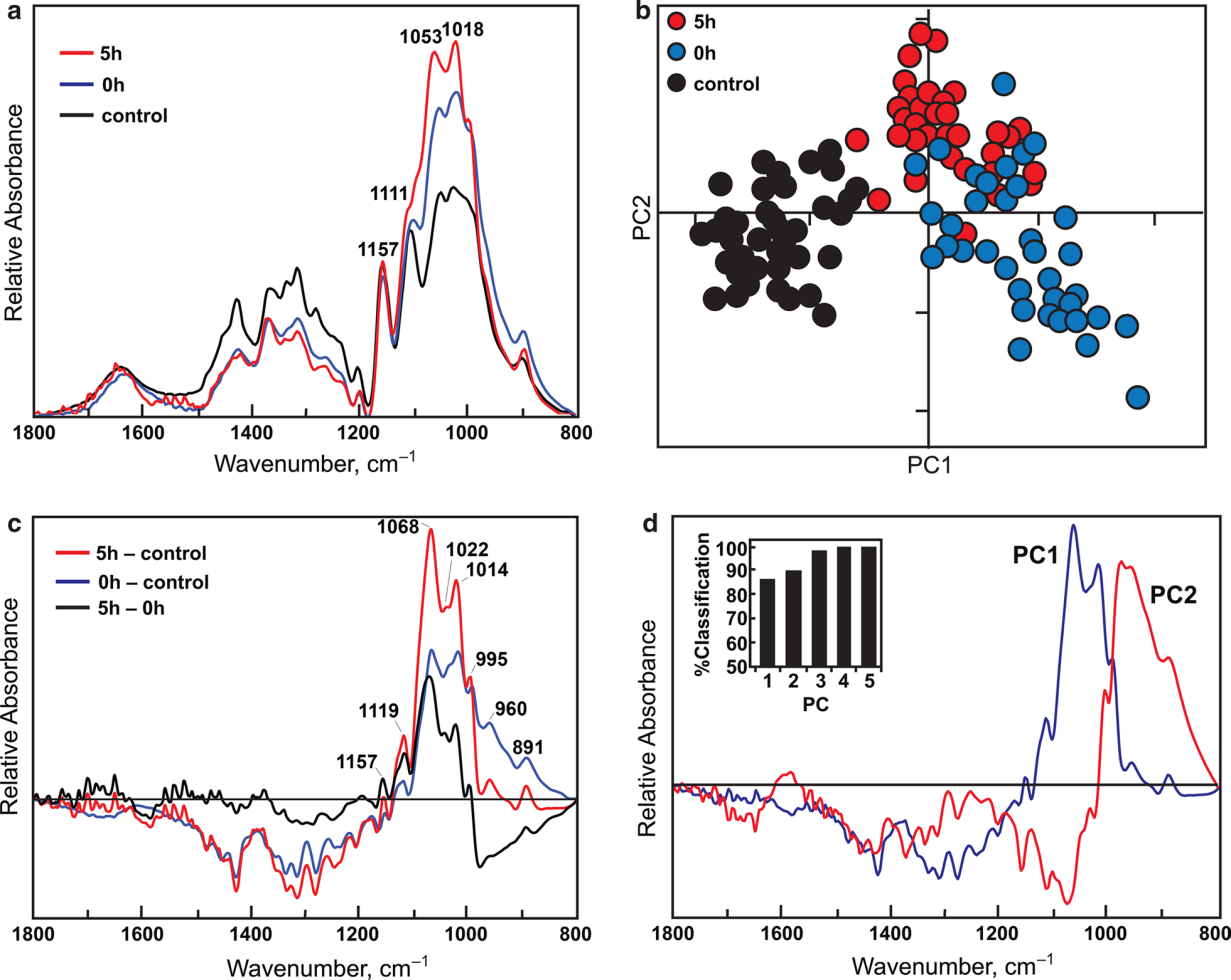
FTIR analysis of crystalline, swollen, and gelatinized states of cotton linter cellulose. **a** Area-normalized averaged spectra of crystalline cellulose standard, and TFA-treated cellulose treated at − 20 °C for 15 h, with and without a subsequent treatment of 55 °C for 5 h. Each spectral line is the average of 25–30 technical replicates. **b** Principal Component Analysis of the individual spectra from each treatment. Each replicate is the sum of 128 co-added FT spectra that were area-normalized. **c** Subtraction spectra of the baseline-corrected and area-normalized averaged spectra shown in **a**. **d** Principal component loadings used in PC shown in **b**. *Inset* Percentage of correct classification of spectra with treatment with increasing PCs

Fourier transform infrared (FTIR) spectra of air-dried cellulose incorporated in KBr disks gave two pronounced peaks at 3411 and 2896 cm^−1^ (Fig. 7), which were assigned to O–H stretches [43]. Absorbances in the O–H stretching region were higher in untreated and TFA-swollen cellulose than in TFA-swollen cellulose heated for 5 h at 55 °C (Fig. 7). The changes in FTIR spectra of gelatinized cellulose indicated a significant reduction in the density of hydrogen bonding within the regenerated cellulose. Absorbances around 1790 cm^−1^ were detected in the gelatinized samples, corresponding to carbonyls of trifluoroacetyl esters formed during heating [44]. However, no carbonyl esters from TFA were detected in 0- or 5-h samples that had been extensively washed and freeze-dried. We used FTIR microspectroscopy to examine differences in the carbohydrate fingerprint region as a result of TFA treatment in the cold and subsequently at 55 °C in freeze-dried materials. Spectra sampled from multiple areas of gold-plated slides were baseline-corrected and area-averaged between wavenumbers 800 and 1800 cm^−1^ before comparisons by digital subtraction or principal components analysis (PCA) (Fig. 8). Thirty spectra obtained from each of three samples (untreated, TFA-swollen cellulose before heating, and TFA-swollen cellulose after heating for 5 h) were averaged and then area-normalized. All three average spectra show peaks characteristic of cellulose at 1157, 1111, 1053, and 1018 cm^−1^. However, the amplitudes of C–C, C–O, and C–H stretches are relatively increased in both TFA-treated samples, indicating increased modes of these molecular vibrations. Digital subtraction of the untreated sample from either the 0 or 5 h samples showed relative enrichment of peaks at 1119, 1068, 1014–1022, 995, 960, and 891 cm^−1^ (Fig. 8b). Carillo et al. (2004) [42] have proposed that a peak at 895 cm^−1^ is diagnostic of crystalline cellulose I and is shifted to 893 cm^−1^ in cellulose II or amorphous cellulose. The peak at 891 cm^−1^ in the digital subtraction spectra may indicate more cellulose II or amorphous cellulose in the TFA-treated samples. Exploratory PCA of all 90 spectra showed that untreated samples could be readily discriminated from treated samples on PC1 (Fig. 8c) and that the loading for PC1 is similar to the digital subtraction spectra (Fig. 8d). Four PCs are sufficient for 100% classification of all three samples (Fig. 8d inset). Digital subtraction of TFA-swollen cellulose with 0 h heating from 5-h heating showed relative enrichment of peaks at 1157, 1119, 1072, and 1022 cm^−1^ that are not characteristic of crystalline cellulose.

**Fig. 9:**
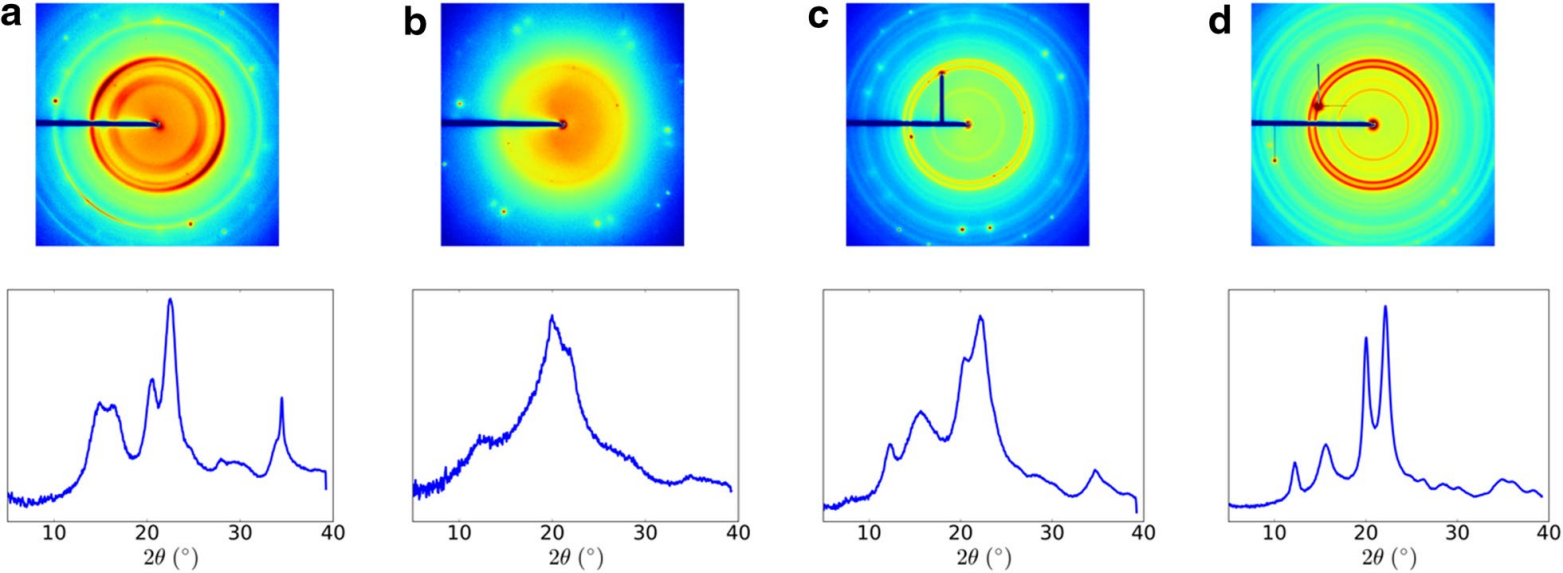
X-ray scattering of control, **a** swollen, **b** gelatinized, and **c** gelatinized and hydrolyzed (2 M TFA at 120 °C, 90 min) cotton linter cellulose. Crystalline cellulose Iβ is converted to cellulose II during gelatinization, precipitation, and freeze-drying

**Fig. 10:**
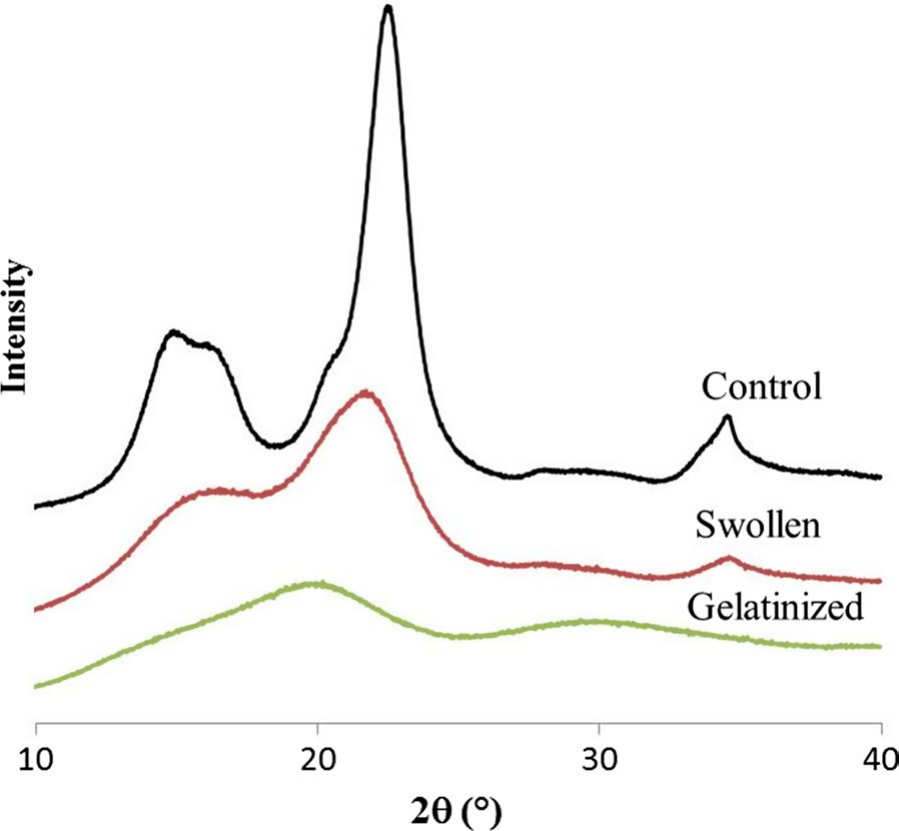
XRD spectra of crystalline, swollen, and gelatinized cellulose. Cotton linter cellulose was low-temperature swollen at − 20 °C for 24 h. Swollen cellulose was gelatinized by heating at 55 °C for 5 h

Loss of crystallinity was also observed by X-ray diffraction, as the sharp crystalline reflections of cellulose Iβ are lost upon dissolution in TFA (Figs. 9 and 10). However, upon freeze-drying X-ray scattering indicates partial recrystallization into cellulose II (Fig. 9). Scattering angles at 2*θ* = 18°, 22.7°, and 34°, corresponding to the (110), (002), and (004) planes of cellulose Iβ [45–47], respectively, were well defined in the cotton linter cellulose but decreased in intensity and shifted in angle upon warming in TFA (Fig. 10). The crystallinity index (CrI) of untreated cellulose, determined as scattering angle of 22.5° relative to that at 18°, was 80%, consistent with the previous XRD analyses [48]. Low-temperature swelling of cellulose in TFA is sufficient to reduce the Segal crystallinity index (CrI) from 80% in the untreated sample to 30% at 0 °C for 2 h and 27% for cellulose swollen at − 15 °C for up to 24 h. When swollen cellulose was heated to 55 °C in TFA, the material became completely amorphous (Fig. 9), and when freeze-dried, the cellulose partially recrystallized as cellulose II (Figs. 9 and 10) with a CrI for cellulose II of 41–58% (2*θ* = 16°, 21.7°).

### Rates of enzymatic hydrolysis of crystalline and gelatinized cellulose

**Fig. 11:**
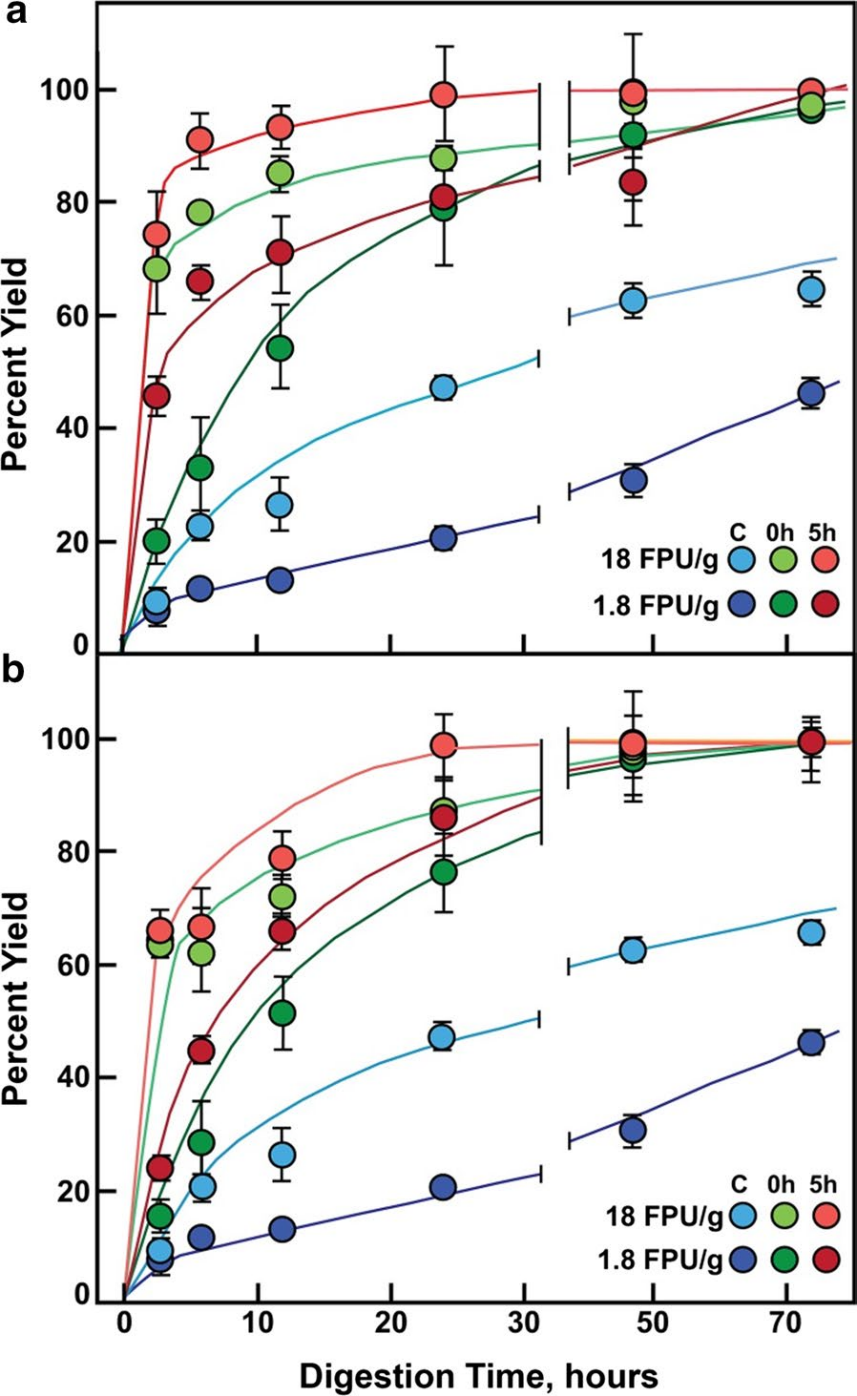
Rates of enzymatic hydrolysis of crystalline (C), swollen (0 h), and gelatinized (5 h) cellulose. Crystalline cellulose was swollen in TFA at − 20 °C for 15 h. Gelatinized cellulose was prepared from swollen cellulose by heating at 55 °C for 5 h. Cellic^®^ Ctec2 (Novozymes) (18 or 1.8 FPU/g glucan) was added to 5 mg of cellulose samples in 2 mL of 50 mM sodium citrate and incubated at 50 °C for up to 72 h. Samples were withdrawn at designated intervals for assay cellulose and solubilized glucose [54]. Panel (**a**) shows samples that were not dried prior to enzymatic hydrolysis. Panel (**b**) shows samples that were freeze dried prior to hydrolysis

Rates of hydrolysis of low-temperature swollen and gelatinized cellulose were substantially enhanced compared to untreated controls (Fig. 11a, b). Never-dried swollen and gelatinized celluloses were completely hydrolyzed by 12 h with cellulase cocktail (1.8 and 18 FPU/g glucan), while less than 50% of control cellulose was digested (Fig. 11a). Control cellulose approached completion only after 72 h. After freeze-drying, swollen cellulose was rendered more resistant to hydrolysis, whereas gelatinized cellulose remained completely hydrolyzed by 12 h (Fig. 11b). This is likely because the freeze-dried samples partially recrystallized to a greatly extent than the samples that were not freeze-dried, as shown by dark field microscopy (Fig. 2). Never-dried swollen and gelatinized cellulose were rapidly digested by moderate amounts of enzyme (18-FPU/g glucan, 15 mg protein/g glucan) to completion between 12 and 24 h, and even low enzyme loading (1.8-FPU/g glucan, 1.5-mg protein/g glucan) resulted in complete digestion by 72 h (Fig. 11a).

### Behavior of crystalline, swollen, and gelatinized cellulose in catalytic conversion to biofuel precursors

**Fig. 12:**
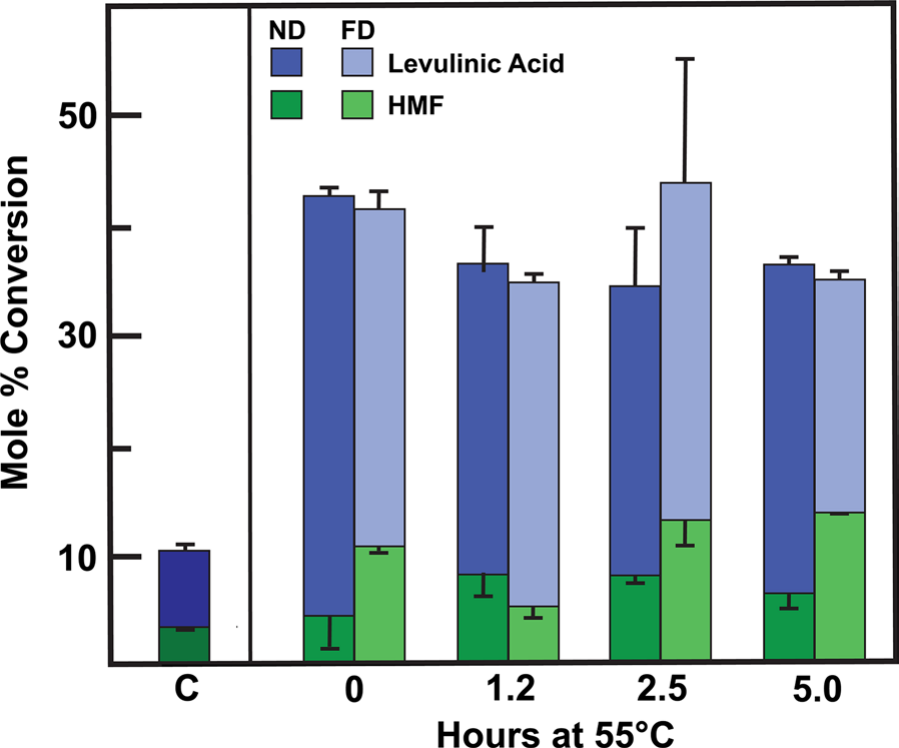
Yields of HMF and levulinic acid from crystalline, swollen, and gelatinized cellulose. Crystalline cellulose was suspended in TFA at − 20 °C for 15 h (0 h), with subsequent heating at 55 °C for up 5 h. Materials (100 mg) were suspended in 2 mL of 100 mM each of maleic acid and AlCl_3_; the reaction mixtures were heated to 180 °C and held at that temperature for 15 min. *ND* never-dried, *FD* freeze-dried. Levulinic acid yield is in blue, and HMF yield is in green. Error bars represent standard deviation for triplicates

Conversion of cotton linter cellulose catalyzed by maleic acid/AlCl_3_ to HMF and levulinic acid was substantially enhanced by TFA-induced conversion of cellulose from crystalline to amorphous form (Fig. 12) [49, 50]. Control experiments with untreated cellulose yielded only about 4 and 5% theoretical yield of HMF and levulinic acid, respectively. However, yields of HMF were at least twofold higher in low-temperature and gelatinized cellulose, with slightly higher amounts in low-temperature swollen but not heated samples (Fig. 12). Yields of levulinic acid were over eightfold higher (41% of theoretical yield) when cellulose swollen at − 15 °C for 24 h was followed by gelatinization. However, cellulose swollen at 0 °C for 2 h with TFA without gelatinization combined for highest HMF yield (14%) and relatively high levulinic acid yield (34.5%) compared to gelatinized samples (Fig. 12).

## Discussion

Low-temperature swelling of cellulose in TFA causes significant disruption of crystallinity, and solubilization at warm temperatures generates mostly amorphous cellulose after gelatinization in ethanol. TFA offers a milder operational temperature range (− 20 to 78 °C) compared to liquid ammonia, and a low boiling point (72.4 °C) enables recovery by distillation. Near anhydrous TFA causes less degradation of sugars compared to other mineral acids [51]. Zhao et al. [37] also reported decrystallization of cellulose when swollen for 3 h at 0 °C and then exposed to a vacuum (30 mTorr) at 105 °C for 2 days. Ethanol gelatinization preserves considerably more cellulose in an amorphous form than does dilution with water to 2 M for hydrolysis (Fig. 5).

As indicated by several physical analyses, changes in crystallinity upon treatment of cellulose with TFA and subsequent gelatinization are biphasic, giving two distinct alterations from crystallinity. Cellulose has a characteristic thermal decomposition profile that peaks between 340 and 360 °C [39]. The dense opaque gels made during low-temperature swelling retain intense birefringence, an indicator of crystallinity, whereas subsequent heating results in gelatinized cellulose whose birefringence becomes diffuse (Fig. 2). The DTG curves of swollen cellulose shift to slightly lower temperatures than untreated cellulose, but fully gelatinized cellulose decomposes at drastically lower temperatures (Fig. 4). The amorphous cellulose decomposition usually appears as a less pronounced shoulder in native cellulose instead of a well-defined peak [40]. Lower crystallinity and shorter cellulose chain length can accelerate the degradation process and reduce the thermal stability [52].

Two distinct states of cellulose from crystalline controls are also observed by principal components analysis of FTIR spectra from low-temperature swollen and gelatinized cellulose (Fig. 8) [53, 54]. Absorbances at 1790 cm^−1^ were detected in fresh heat gelatinized samples and correspond to the carbonyl group of trifluoroacetyl ester groups [44]. Part of this behavior might be attributed to two different interactions with TFA. While TFA diesters are suspected of disrupting crystallinity at cold temperatures, cellulose can be selectively trifluoroacetylated at the C6-hydroxyl groups in the TFA solution above ambient temperature (Fig. 7) [37, 44]. However, subsequent washing of the gelatinized cellulose with 80% ethanol in water (v/v) and then water results in loss of these esters, as detection of absorbance at 1790 cm^−1^ by these carbonyl esters is lost upon subsequent freeze-drying (Fig. 8). Taken together, the increased amplitudes in carbohydrate stretching observed in FTIR spectra and the X-ray diffraction data indicate that TFA treatment decreases cellulose crystallinity. Although some crystallites reform upon freeze-drying, these may be predominantly cellulose II crystals rather than cellulose I.

X-ray diffraction also indicates a loss of crystallinity of the cellulose during incubation in TFA at low temperature followed by conversion to cellulose II upon heating (gelatinization), precipitation, and freeze-drying (Fig. 9). The major peak of untreated and swollen cellulose is around 21.7° (Figs. 9 and 10). Gelatinized and freeze-dried cellulose show recrystallization as cellulose II. The significant structure changes could be traced by 2*θ* variation of (1 − 1 0) and (1 1 0). This is more clearly shown by XRD of residual crystalline cellulose after hydrolysis of the gelatinized cellulose by TFA at 120 °C for 90 min (Figs. 5a and 9c). The 2*θ* of (1 1 0) for cellulose II is 19.9°. A shoulder peak appears near to (1 1 0), that is (2 0 0) at 22.1°. The transition of cellulose Iβ to cellulose II gives rise to the shift of (1 − 1 0) from 15° to 12.2° and (1 1 0) shifts from 16.5° to 19.9°, very close to (2 0 0) [46, 47, 55, 56].

The two physical states of low-temperature swollen and gelatinized cellulose also result in altered degrees of resistance to acid hydrolysis. While 20% of untreated cellulose is hydrolyzed by hot 2-M TFA, an additional 30% of total mass is hydrolyzed in the dense opaque swollen cellulose, and yet, another 20% is hydrolysis in fully gelatinized cellulose (Fig. 5). In contrast, enzymatic digestion is enhanced substantially by low-temperature swelling (Fig. 11). Commercial cellulase was able to completely hydrolyze swollen cellulose within 72 h, even at the low loading of 1.8-FPU/g cellulose. Gelatinized cellulose, formed after heating at 55 °C, hydrolyzed at only a slightly faster rate. Untreated cellulose was 80% digested at the highest enzyme loading and only 40% digested at the lowest after 72 h.

Taken together, these data indicate that regenerated cellulose has more accessible cellulose compared to cellulose swollen at 0 °C, because levulinic acid is produced from HMF, which must first be made from the glucose hydrolyzed from the cellulose. A similar trend was observed in samples chilled to − 15 °C for 24 h with and without a dissolution step, but with a more significant levulinic acid difference in yield. All regenerated cellulose resulted in a higher levulinic acid yields compared to swollen cellulose. However, the differences between swollen and regenerated cellulose are far smaller than the difference between untreated cellulose and any treatment of cellulose with TFA. These data indicate that mild decrystallization of cellulose results in significantly more reactive cellulose for hydrolysis and conversion of the resultant glucose using acid catalysts.

Operating industrial-scale processes at 0 °C will require significant energy input for heat removal. However, the low boiling point of TFA allows for recovery of rejected waste heat from downstream cellulose conversion processes, which may represent a significant cost savings. Detailed economic analysis was beyond the scope of the work reported here. Additional efforts to examine this technology should focus on comparative techno-economic analyses with ionic liquid and other advanced pretreatments to clearly identify where cost savings can have the greatest impact.

## Conclusions

Low-temperature swelling of cellulose TFA followed by gelatinization at warm temperatures generates amorphous cellulose that is readily hydrolyzed by acid. While low-temperature swelling retains significant crystalline structure, the alteration of crystallization alone is sufficient to significantly enhance both the enzymatic digestion and maleic acid/AlCl_3_-catalyzed conversion of cellulose to levulinic acid and HMF. A closed system of swelling cellulose in TFA and recovery in ethanol represents a cost-effective pretreatment that markedly enhances enzymatic hydrolysis and catalytic conversion to biofuel intermediates. Distillation and separation of TFA and ethanol allow safe regeneration and recycling of the TFA for continuous generation of modified cellulose.

## Methods

### Materials

Cotton linter cellulose (Sigmacell; product No. S5504T), maleic acid, and AlCl_3_·6H_2_O were purchased from Sigma-Aldrich. Trifluoroacetic acid (TFA, 99%) was purchased from Alfa Aesar and Sigma-Aldrich.

### Cellulose swelling and gelatinization

Cotton linter cellulose was mixed thoroughly with ice-cold 99% TFA at 50 mg mL^−1^ in borosilicate glass tubes sealed with Teflon^®^-lined caps or scaled to 1-g preparations suspended in 30 mL of TFA in 50-mL Falcon tubes. Suspensions were placed at 0, − 15, or − 20 °C for up to 24 h before incubation at 55 °C for up to 5 h. After treatment, four volumes of ethanol were added with rapid vortex mixing to gel and precipitate the cellulose. The ethanol-precipitated gels were pelleted by centrifugation, and the supernatant liquid was transferred to a 4-mL screw-cap vial, 0.5 mL of *t*-butyl alcohol added to prevent decomposition, and the mixture was dried under a stream of warm air. The gels were washed with additional 80% ethanol in water, and this added to the supernatant fraction. The gels were washed with three times with water and either brought to 5-mg mL^−1^ water and stored at 4 °C (never-dried), or freeze-dried. For scaled up reactions, two volumes of ethanol were used to rinse the contents of the 50 mL Falcon tubes into a 250-mL beaker with rapid stirring to produce the cellulose gels. The cellulose gels were collected on glass-fiber filter disks (Whatman GF/D) and washed five times with four volumes of 80% ethanol to remove residual TFA. The filter cakes were resuspended in deionized water and then freeze-dried.

### Darkfield and differential interference contrast microscopy

Cellulose samples were placed on glass microscope slides without additional treatment or staining. Images were captured using a Nikon C1 Plus microscope (Nikon, Tokyo, Japan) configured for either darkfield or differential interference contrast (DIC) illumination and using a SPOT RTKE CCD camera (Diagnostic Instruments, Sterling Heights, MI). FIJI (ImageJ) was used to rotate, crop, normalize brightness, and convert 16-bit color images to 8-bit grayscale images.

### Scanning electron microscopy (SEM)

The surface morphology of cellulose samples was examined using a Hitachi S3400N (Tokyo, Japan) microscope with an accelerating voltage of 15 kV. Images showing surface morphologies of the cellulose were taken at 100 and 5000 magnifications. Before examination, a fine layer of gold was sprayed on samples by an ion sputter coater with a low deposition rate.

### Thermogravimetric analysis (TGA)

Thermogravimetric analysis was performed on a SDT Q600 from TA Instruments (New Castle, DE USA) under Nitrogen flow (50 mL min^−1^). The samples weighing approximately 5 mg were packed in aluminum pans. The samples were tested from the ambient temperature to 700 °C at a heating rate of 20 °C min^−1^.

### Hydrolysis of crystalline, swollen, and gelatinized cellulose

Samples (1 mg) of dry cellulosic materials or in 0.5 mL aqueous never-dried suspensions in 4-mL borosilicate glass vials were brought to 2-M TFA containing 500 nmoles of *myo*-inositol (internal standard), sealed with Teflon^®^-lined screw caps, and heated to 120 °C for 90 min with occasional shaking. After cooling, the remaining insoluble material was pelleted by centrifugation at 2500×*g* for 5 min. The clear supernatant liquid was transferred to a 4-mL glass vial and dried under a stream of N_2_ at 45 °C. The pellet was washed twice with water followed by centrifugation, and suspended in 0.8 mL of water. Samples were assayed for glucose equivalents by phenol–sulfuric assay [57].

To determine monosaccharide distribution, dried soluble fractions were hydrolyzed in 1 mL of 2-M TFA at 120 °C for 90 min, then 0.5 of *tert*-butyl alcohol was added, and the mixed was dried under a stream of nitrogen at 45 °C. A portion of the dried hydrolyzates were reduced with NaBH_4_ and 1-methylimidazole-catalyzed acetylated as described previously [41]. Alditol acetates of the monosaccharides recovered were identified and quantified by GLC–MS compared to the *myo*-inositol internal standard wall material. Derivatives were separated on a 0.25-mm × 30-m column of SP-2330 (Supelco, Bellefonte, PA). Temperature was held at 80 °C during injection, then ramped quickly to 170 °C at 25 °C min^−1^, and then to 240 °C at 5 °C min^−1^ with a 10 min hold at the upper temperature. Helium flow was 1 mL min^−1^ with splitless injection. Derivative structures were confirmed by electron-impact mass spectrometry [58].

### Light-scattering determinations of relative molecular size

Crystalline and amorphous celluloses were mixed in about 3 g of the ionic liquid 4-methylmorpholino-4-oxide hydrate (NMMO) and heated for 4 h at 90 °C to produce a homogeneous molten mixture that was brought to 10 mg mL^−1^ final concentration. Light scattering measurements (Malvern Zetasizer DL, source) were made in 1 cm disposable cuvettes heated to 85 °C.

### Fourier transform infrared (FTIR) spectroscopy

The structural changes of untreated and TFA-treated cellulose were investigated by FTIR spectroscopy using a Perkin Elmer Spotlight 400 FTIR spectrometer (Perkin Elmer, Waltham, MA, USA). The samples were oven dried at 105 °C for 5 h, mixed with KBr in a ratio of 1:200 (w/w), and pressed under vacuum to form pellets. The FTIR spectrum of the samples was recorded in the transmittance mode in the range of 4000–500 cm^−1^ at a spectral resolution of 4 cm^−1^ and 64 scans per sample.

FTIR microspectroscopy on dry cellulosic materials was essentially as described by McCann et al. [25]. Briefly, materials mounted in the wells of IR-reflective, gold-plated microscope slides (Thermo-Electron) were placed on the stage of a Nicolet Continuum series microscope accessory to a 670 IR spectrophotometer with a liquid nitrogen-cooled mercury–cadmium telluride detector (Thermo-Electron). Spectral collection in transflectance mode was made on cellulosic material within a 125 × 125 μm window. In transflectance, the beam is transmitted through the wall sample, reflected off the gold-plated slide, and then transmitted through the sample a second time. Spectra were co-added from 128 A collected with 8-cm^−1^ resolution. Each individual co-added spectrum from each sample was then area-averaged and baseline-corrected. Spectra from up to 30 samples were then averaged and used for digital subtraction.

Baseline-corrected and area-normalized data sets of spectra were then used in the chemometric analyses. The PCA was carried out with the WIN-DAS software [59]. LDA was used to develop a discriminative calibration model to classify spectra into groups. The distances between each observation were estimated from group centers. Mahalanobis distance was used as the distance metric [59] to measure the distance of each observation (spectrum) from each group center. LDA using squared Mahalanobis distance metrics was applied to the PCA scores of original data [59]. The derived quantities such as group centers and covariance matrices were calculated from the transformed observations, and the assignments to the respective class were then made.

### X-ray diffraction (XRD)

The structural analysis of the samples was evaluated by X-ray diffraction (XRD) using a LabX XRD-6000 (Shimadzu, Kyoto, Japan) diffract meter with Cu Kα radiation source (*λ* = 1.54060 Å). The XRD patterns were obtained over the angular range 2*θ* = 10–40^o^ in 0.04 degree per step. The empirical method proposed by Segal et al. [60] was used to calculate the crystalline index (CrI) of cellulose I in respect of 002 plane:

$${\text{CrI }} = \left( {\left( {I_{002} - I_{\text{am}} } \right)/I_{002} } \right) \times 100,$$

where CrI is the crystallinity index, *I*_002_ is the maximum intensity of the (002) peak at 2*θ* = 22.7^o^, and *I*_am_ is the intensity at 2*θ* = 18.0^o^. Additional X-ray patterns were collected using a 5-μ X-ray beam at GM/CA, beamline 23ID-B at APS at Argonne National Laboratory [61] with a sample to detector distance of 300 mm and X-ray wavelength of 1.033 Å. Calculation of 2*θ* for data from the GM/CA beamline (1.033 Å) was corrected to 1.54060 Å for comparison purposes in Fig. 9. The sharp reflections within diffraction patterns come from mica membrane onto which samples were mounted for experiment. These reflections had been masked out for the calculation of circularly averaged intensity.

### Catalytic conversion of regenerated cellulose to HMF and levulinic acid

The native and regenerated cellulose were hydrolyzed and the resultant glucose was sequentially converted to HMF, and levulinic and formic acids using maleic acid and aluminum chloride as catalysts. Reaction procedures were previously reported by Zhang et al. [50, 62].

### HPLC analysis

The concentrations of glucose, fructose, HMF, levulinic, and formic acids were analyzed by a Waters HPLC system, equipped with a Waters 1525 pump and Waters 2412 Refractive Index detector (Waters, Milford, MA). An HPX-87H AMINEX column (BioRAD, Hercules, CA) was used for separation with 5-mM aqueous H_2_SO_4_ and 5% (w/w) acetonitrile as the mobile phase at a flow rate of 0.6 mL/min. The acetonitrile was used to facilitate the separation of hexoses and maleic acid [63, 64]. The column temperature was maintained at 338 K. All concentrations of sugars and organic products in the aqueous phase were determined by external calibration standards.

### Enzymatic hydrolysis of cellulose

Enzymatic hydrolysis experiments were performed with 5 mg suspended in 2 ml of 50-mM sodium citrate buffer, pH 5.0, containing 1 or 0.1-µL Cellic™ Ctec2 (18 or 1.8 FPU/g cellulose (corresponding to 15- or 1.5-μg protein/mg glucan) for TFA-treated and untreated cellulose. Enzymatic hydrolysis was carried out at 50 °C in a rolling hybridization oven at 250 rpm. Each experiment was performed in duplicate. During hydrolysis, samples were taken at predetermined intervals for analysis using HPLC. Remaining cellulose and soluble sugar were separated by centrifugation, and glucose equivalents in each were determined by a phenol–sulphuric assay [54].

### Statistics analysis

All data collected were subject to the analysis of variance ANOVA (*P* < 0.05) using SPSS. All the analyses were carried out in duplicates.

## Abbreviations

TFA: trifluoroacetic acid
HMF: 5-hydroxymethylfurfural
HPLC: high-performance liquid chromatography
FTIR: Fourier transform infrared
XRD: X-ray diffraction
SEM: scanning electron microscopy
CrI: crystallinity index
DP: degree of polymerization
TGA: thermogravimetric analysis
DTG: derivative thermogravimetry
ANOVA: analysis of variance
SPSS: software package used for statistical analysis
